# Analysis of an Outbreak of Puerperal Fever Due to Group A Streptococci by Random Amplified Polymorphic DNA Fingerprinting

**DOI:** 10.1155/S1064744997000392

**Published:** 1997

**Authors:** Jacques F. G. M. Meis, Harry L. Muytjens, Paul P. van den Berg, Andreas Voss, Willem J. G. Melchers

**Affiliations:** 1 Department of Medical Microbiology MMB 440 P.O. Box 9101 University Hospital Nijmegen Nijmegen 6500 HB The Netherlands; 2 Department of Obstetrics and Gynecology University Hospital Nijmegen Nijmegen The Netherlands

## Abstract

*Objective: Streptococcus pyogenes* is the cause of the classical childbed fever and can occur in both sporadic and epidemic form. Once an outbreak is identified on a maternity ward it is not only necessary to place the patients in strict isolation but also identify to the source of the infection. Fast reliable typing methods can aid in infection control.

*Methods:* An outbreak of puerperal fever due to *S. pyogenes* was analyzed by random amplified polymorphic DNA (RAPD) analysis.

*Results:* Identical fingerprint patterns were found in isolates of 3 patients, the throat and infected finger of the delivering obstetrician, 2 of the physician's family members, and from the cervix of a woman who was examined by the physician 7 months after the outbreak. The outbreak was stopped after antimicrobial treatment of the physician and his family members.

*Conclusions:* RAPD typing appeared to be a fast and reliable tool for epidemiological studies of *S. pyogenes* and is probably more efficient in strain differentiation than classical M and T serotyping.


Infectious Diseases in Obstetrics and Gynecology 5:232–236 (1997)
© 1997 Wiley-Liss, Inc.

### Analysis of an Outbreak of Puerperal Fever Due to Group A Streptococci by Random Amplified Polymorphic DNA Fingerprinting

**Jacques F.G.M. Meis,[1]* Harry L. Muytjens,[1] Paul P. van den Berg,[2] Andreas Voss,[1] and Willem J.G. Melchers[1]**

[1]*Department of Medical Microbiology, University Hospital Nijmegen, Nijmegen, The Netherlands*
[2]*Department of Obstetrics and Gynecology, University Hospital Nijmegen, Nijmegen, The Netherlands*

### Abstract

***Objective:*** ***Streptococcus pyogenes*** **is the cause of the classical childbed fever and can occur in both sporadic and epidemic form. Once an outbreak is identified on a maternity ward it is not only necessary to place the patients in strict isolation but also identify to the source of the infection. Fast reliable typing methods can aid in infection control.**

***Methods:*** **An outbreak of puerperal fever due to** ***S. pyogenes*** **was analyzed by random amplified polymorphic DNA (RAPD) analysis.**

***Results:*** **Identical fingerprint patterns were found in isolates of 3 patients, the throat and infected finger of the delivering obstetrician, 2 of the physician's family members, and from the cervix of a woman who was examined by the physician 7 months after the outbreak. The outbreak was stopped after antimicrobial treatment of the physician and his family members.**

***Conclusions:*** **RAPD typing appeared to be a fast and reliable tool for epidemiological studies of** ***S. pyogenes*** **and is probably more efficient in strain differentiation than classical M and T serotyping. Infect. Dis. Obstet. Gynecol. 5:232–236, 1997.** © 1997 Wiley-Liss, Inc.

Key words

*Streptococcus pyogenes;* genotyping; maternity ward

Streptococcus pyogenes [group A beta hemolytic *Streptococcus* (GAS)] has been identified as the cause of a number of infectious diseases, including pharyngitis, scarlet fever, impetigo, erysipelas, puerperal fever, septicemia, and toxic shock-like syndrome as well as the non-suppurative sequelae rheumatic fever and acute glomerulonephritis. In the last decade an increase in the number of severe infections due to GAS has been noted.[1] GAS is an important pathogen causing outbreaks of infections in newborns,[2] institutionalized elderly persons,[3] and classically, puerperal woman.[4–8] Puerperal infections due to GAS were, before the availability of penicillin, a common and lethal nosocomial scourge. Despite a drastic decrease in the incidence of these infections, epidemics are still regularly described and most often related to a member of the hospital staff. Epidemiological investigations of these outbreaks have almost entirely focused on the analysis of cell surface proteins for serotyping. However, classical serological M typing for strain classification is available only in a few reference laboratories worldwide, while T typing offers lower discriminatory power. Therefore, several alternative genotypic methods have been applied to differentiate GAS isolates, such as multilocus enzyme

*Correspondence to: Dr. Jacques F.G.M. Meis, Department of Medical Microbiology, MMB 440, P.O. Box 9101, University Hospital Nijmegen, 6500 HB Nijmegen, The Netherlands. E-mail:j.meis@mmb.azn.nl

Received 3 March 1997
Accepted 23 June 1997

Clinical Study electrophoresis,[9] restriction fragment analysis of total DNA,[10] ribotyping,[10] and pulsed-field gel electrophoresis.[11] Recently, random amplified polymorphic DNA (RAPD) analysis has been recommended for the typing of GAS.[12] Because this technique is a simple and easy way to characterize GAS isolates, we applied it to investigate an outbreak of puerperal fever due to GAS in our maternity ward.

### SUBJECTS AND METHODS

The outbreak involved 3 postpartum women as well as their delivering obstetrician. All women had a vaginal delivery. None of the infants developed signs of infection or were colonized.

### Case Reports

#### *Patient 1*

A 36-year-old woman, gravida I para 0, was admitted to our hospital in the 40th week of gestation because of pregnancy-induced hypertension. The same day she had a vaginal delivery of a healthy boy of 3,865 g. On the 3rd day postpartum the uterus became enlarged and tender and the temperature rose to 40°C. Many gram-positive cocci were noted in a Gram-stained smear of a cervical swab. Despite immediate treatment with intravenous amoxicillin, the patient became critically ill with chills and hypotension and was admitted to the intensive care unit for circulatory support. Antimicrobial therapy was changed to cefuroxime, gentamicin, and metronidazole. The latter two drugs were discontinued when the cervical culture grew GAS. Blood cultures remained negative. The patient became afebrile after 2 days of treatment and was discharged 8 days later in good clinical condition.

#### *Patient 2*

A 26-year-old woman, gravida I para 0, was admitted in the 37th week of pregnancy. The same day she had a spontaneous delivery of a boy of 3,125 g. The 1st day postpartum she became feverish (38.1°C) and the temperature rose to 40°C on the 3rd day postpartum. There was uterine tenderness on palpation but an ultrasound examination showed that the cavum uteri was empty. A few gram-positive cocci were observed in a Gram-stained smear of the cervix. Treatment was started empirically with cefuroxime, gentamicin, and metronidazole. The latter two drugs were stopped when the cultures of the cervix and urine grew GAS the next day. Blood cultures remained negative. The patient was afebrile 3 days after the start of antimicrobial treatment and was discharged in good clinical condition 7 days postpartum.

#### *Patient 3*

A 30-year-old woman, gravida II para I, was admitted in the 43rd week of pregnancy. Priming of the cervix was performed with prostaglandins, after which she went into labor and delivered a healthy boy of 3,865 g. On the 2nd postpartum day she developed fever (38°C), lower abdominal pain, and uterine tenderness on palpation. Ultrasound examination of the uterus revealed an empty cavum. A predominance of gram-positive cocci was noted in a Gram-stained smear of the cervix. Antimicrobial treatment was started with intravenous amoxicillin, which was changed to cefuroxime because of persistent fever. Cervix and urine cultures grew GAS. Blood cultures remained negative. The patient became afebrile after 4 days of treatment and was discharged 1 day later with oral amoxicillin.

All 3 patients delivered their children in 3 consecutive days with the assistance of the same obstetrician who was possibly the source of the outbreak. He appeared to have had a sore throat for 3 days and noticed an infection along the nail of his middlefinger on the day of onset of the first puerperal fever. GAS was cultured from his pharynx and the infected skin lesion of the finger as well as from the throat of 2 asymptomatic children in his family. The obstetrician as well as the culture-positive members of his family were treated orally with amoxicillin for 10 days. Repeated throat cultures after the treatment period were negative. Other sites were not cultured. Eleven months after this outbreak of puerperal fever, GAS was isolated from the cervix of another non-puerperal woman with complaints of vaginal discharge. She was examined by the index case 7 months after the outbreak. Throat swabs were culture negative.

### Bacterial Isolates

Identification of beta hemolytic colonies was done with commercial latex-agglutination kits (Streptex, Murex Diagnostics, Utrecht, The Netherlands).

Clinical isolates of *S. pyogenes* from 3 patients, the physician and family contacts, and sporadic

TABLE 1. Summary of GAS isolates involved in the outbreak and control isolates

| Lane | Specimen | Date of sampling (day/month/ year) | T type | RAPD type |
|---|---|---|---|---|
| 1 | Throat swab | 6-12-92 | ND[a] | I |
| 2 | Bronchial secretion | 9-02-93 | ND | II |
| 3 | Throat swab | 19-03-93 | ND | III |
| 4 | Pus, hand | 14-07-93 | ND | IV |
| 5 | Cervix swab | 17-11-92 | 11 | V |
| 6 | Pus, abdominal wound | 18-06-93 | ND | VI |
| 7 | Pus, head wound | 12-11-93 | ND | VII |
| 8 | Pus, leg wound | 23-11-93 | ND | VIII |
| 9 | Throat swab | 27-12-91 | ND | IX |
| 10 | Blood | 20-11-93 | ND | X |
| 11 | Urine, case 2 | 20-12-91 | 11 | V |
| 12 | Throat, index case | 21-12-91 | 11 | V |
| 13 | Pus finger, index case | 20-12-91 | 11 | V |
| 14 | Cervix, case 3 | 20-12-91 | 11 | V |
| 15 | Urine, case 3 | 20-12-91 | 11 | V |
| 16 | Cervix, case 1 | 19-12-91 | 11 | V |
| 17 | Cervix, case 2 | 20-12-91 | 11 | V |
| 18 | Throat, contact 1 | 27-12-91 | 11 | V |
| 19 | Throat, contact 2 | 27-12-91 | 11 | V |
| 20 | Nose, contact 2 | 27-12-91 | 11 | V |

[a]ND = not determined.

clinical isolates from 9 patients presenting with pharyngitis, infected wounds, pneumonia, and sepsis were used in the study (Table 1). One strain was isolated from a vaginal swab of a patient with fluor vaginalis 11 months after the outbreak had occurred. The outbreak strains and strains from the physician and his family members were T serotyped at the National Streptococcal Reference Laboratory.

### Molecular Epidemiology

### *Extraction of Bacterial DNA*

All *S. pyogenes* isolates were grown in Todd-Hewitt broth. Genomic DNA for RAPD was prepared as follows: cultures were centrifuged and washed twice in phosphate buffered saline, resuspended in 250 µl STET buffer [233 mM sucrose, 50 mM Tris-HCl (pH 8.0), 20 mM EDTA, 5% Triton X-100]. Lysozyme was added to a final concentration of 1.7 mg/ml. The suspension was incubated at room temperature for 5 min, heated at 100°C for 1 min, and put on ice for another 2 min. In succession, sodium dodecyl sulfate and proteinase K were added to the solution to a final concentration of 0.3% and 0.5 mg/ml, respectively, which was then incubated at 55°C for 2 h. Following extraction with phenol, 0.03 mg/ml RNase A was added and the mixture was incubated at 37°C for 20 min. The solution was extracted successively with phenol/chloroform/isoamylalcohol (25:24:1) and chloroform/isoamylalcohol (24:1). DNA was precipitated overnight and resuspended in 100 µl of distilled water. An aliquot was electrophoresed in a 1% agarose gel containing 0.1 µg/ml ethidium bromide to estimate the DNA yield and verify DNA integrity.

### *RAPD Analysis*

Polymerase chain reaction (PCR) fingerprinting of *S. pyogenes* DNA (50 ng) was performed in a 50 µl reaction volume containing 75 mM Tris-HCl (pH 9.0), 2.5 mM $MgCl_2$, 20 mM $(NH4)_2SO_4$, 0.01% Tween-20, 0.2 mM dNTPs each, 50 pmol of primer 1283 (5′-GCGATCCCCA-3′),[13] and 0.2 U of Taq DNA polymerase (Thermoperfectplus DNA polymerase, Integro, Zaandam, The Netherlands). A Perkin-Elmer 9600 thermocycler was used for amplification. The cycling program run was 4 cycles of 94°C for 5 min, 36°C for 5 min, 72°C for 5 min, followed by 30 cycles of 94°C for 1 min, 36°C for 1 min, 72°C for 2 min, and a 10 min incubation at 72°C.[13] Amplified DNA (5 µl) was separated by electrophoresis in 1.5% agarose gels and visualized by ethidium bromide staining (0.1 µg/ml). A molecular size marker (100 bp ladder; Pharmacia, Uppsala, Sweden) was used for reference. Gels were photographed and banding patterns were interpreted by visual inspection. PCR fingerprinting was performed with 13 different RAPD primers as described previously.[14] Primer 1283[13] gave the highest resolution and was therefore used for the final analysis.

### RESULTS

The presented cases were the only 3 patients with puerperal fever at the maternity unit documented to have GAS infection. The infection developed on 3 consecutive days. No other cases of puerperal fever due to GAS had been identified in our hospital in the last 10 years before this outbreak. It became obvious that the obstetrician who cared for all 3 patients was the source, once his finger became infected. He did wear surgical gloves, but no mask during the deliveries. The outbreak strains and strains from the physician and his family were all serotype T 11. Identical RAPD patterns were found among the isolates of the patients, the throat

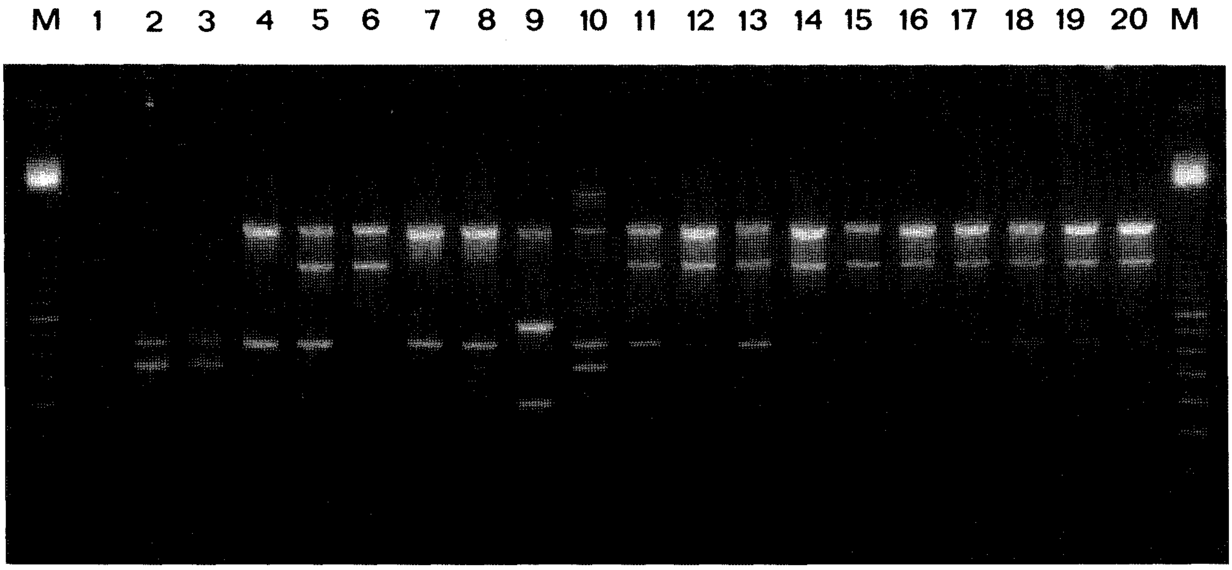

Fig. 1. RAPD typing patterns of *S. pyogenes* isolates involved in an outbreak of puerperal fever: contacts and index case **(lanes 11–20)** and control isolates **(lanes 1–10).** Lane numbers correspond with individual isolates as described in Table 1. M = size marker (100 bp ladder).

and finger of the obstetrician, and the throat and nose isolates of 2 of his children (Fig. 1). The 9 epidemiologically unrelated clinical isolates generated clearly different RAPD patterns (Fig. 1). One culture from a vaginal swab 11 months after the outbreak appeared to be identical to the outbreak strains.

### DISCUSSION

Major epidemics of puerperal infections with GAS occurred until the beginning of this century but were gradually controlled due to better hygienic measures, especially handwashing and the availability of penicillin. In modern times, postpartum infections with GAS are uncommon and most of the puerperal infections are now caused by endogenous bacteria such as beta hemolytic streptococci non-group A, anaerobes, coliforms, and genital mycoplasmas.[15] Whenever one or more postpartum patients develop fever due to GAS, the source should be immediately sought. In most outbreaks, the source appeared to be an asymptomatically colonized health care worker or a mild case of infection of the throat,[5] skin,[8] or anus.[7,16] Rarely the source of infection is traced to environmental contamination.[4] Endogenous infection from nasal carriership results only in sporadic cases. Outbreak investigations should include pharynx, nose, and any abnormal skin cultures as well as anal and vaginal swabs. The umbilicus of the infant can become colonized and reinfect the mother.[6] The same holds true for members of the household of a health care worker who can retransmit the organism.

Earlier, a health care worker with a finger wound due to GAS acted as a source of an outbreak.[8] Although transmission through non-intact gloves might be possible, it seems most likely that GAS in the cases described herein was spread from the physician's sore throat and not from his infected finger, which was covered by surgical gloves. The infection of his finger became apparent after he had delivered all patients and the first patient already had developed puerperal fever. No obvious breaks in standard infection control practices were identified.

If more than one wound isolate of GAS is cultured, all isolates should be typed to identify common patterns. The value of typing has been stressed by several authors and is again demonstrated by the fact that the single isolate cultured from the cervix of a woman 11 months after the outbreak belonged to the same T serotype and RAPD genotype as the outbreak isolates. The only contact between this woman and the obstetrician involved in the outbreak was an examination 7 months after the outbreak. Although it is not clear whether there is a causal relationship, outbreaks associated with the same health care worker can be separated as far as 14 months.[16] Unfortunately, it is not known whether the physician was an anal carrier of GAS or remained an anal carrier after treatment. Therefore, we also recommend anal sampling in the investigation of an outbreak of GAS, even when the source (throat and finger) seems so obvious.

### CONCLUSIONS

This outbreak demonstrates the ability of mildly infected or colonized health care workers with GAS to cause a rapid spread of puerperal fever. The occurrence of a single case of GAS in a maternity ward should immediately trigger a search for a source and more possible cases. The usual typing of T and M antigens for epidemiologic investigation of outbreaks can be hampered by the widespread occurrence of a common serotype or nontypeable isolates. We and others[12] have shown that RAPD analysis does not have this drawback, and we recommend it as a fast and simple tool for routine use in infection control.

### REFERENCES

1. Demers B, Simon AE, Vellend H, et al.: Severe invasive group A streptococcal infections in Ontario, Canada: 1987–1991. Clin Infect Dis 16:792–800, 1993.
2. Isenberg HD, Tucci V, Lipsitz P, Facklam RR: Clinical laboratory and epidemiological investigations of a *Streptococcus pyogenes* cluster epidemic in a newborn nursery. J Clin Microbiol 19:366–370, 1984.
3. Schwartz B, Elliot JA, Butler JC, et al.: Clusters of invasive group A streptococcal infections in family, hospital, and nursing home settings. Clin Infect Dis 15: 277–284, 1992.
4. Claesson BEB, Claesson ULE: An outbreak of endometritis in a maternity unit caused by spread of group A streptococci from a showerhead. J Hosp Infect 6:304–311, 1985.
5. Exalto N, Snethlage W, Buiting HPJ: De epidemische vorm van kraamvrouwenkoorts (in Dutch with English summary). Ned Tijdschr Geneeskd 122:892–897, 1978.
6. McGregor J, Ott JA, Villard M: An epidemic of "child bed fever." Am J Obstet Gynecol 150:385–388, 1984.
7. McIntyre DM: An epidemic of *Streptococcus pyogenes* puerperal and postoperative sepsis with an unusual carrier site—the anus. Am J Obstet Gynecol 101:308–314, 1968.
8. Torbet TE, Porter IA: *Streptococcus pyogenes* infection in a maternity hospital. Health Bull 17:11–12, 1969.
9. Musser JM, Gray BM, Schlievert PM, Pichochero ME: *Streptococcus pyogenes* pharyngitis: Characterization of strains by multilocus enzyme genotype, M and T protein serotype, and pyrogenic exotoxin gene probing. J Clin Microbiol 30:600–603, 1992.
10. Seppala H, Vuopi-Varkila J, Osterblad M, et al.: Evaluation of methods for epidemiologic typing of group A streptococci. J Infect Dis 169:519–525, 1994.
11. Martin PR, Single LA: Molecular epidemiology of group A *Streptococcus* M type 1 infections. J Infect Dis 167: 1112–1117, 1993.
12. Seppala H, He Q, Osterblad M, Huovinen P: Typing of group A streptococci by random amplified polymorphic DNA analysis. J Clin Microbiol 32:1945–1948, 1994.
13. Akopyanz N, Bukanov NO, Westblom T, Kresovich S, Berg DE: DNA diversity among clinical isolates of *Helicobacter pylori* detected by PCR-based RAPD fingerprinting. Nucleic Acids Res 20:5137–5142, 1992.
14. Heuvelink AE, Van de Kar NCAJ, Meis JFGM, Monnens LAH, Melchers WJG: Characterization of verocytotoxin-producing *Escherichia coli* O157 isolates from patients with haemolytic uraemic syndrome in Western Europe. Epidemiol Infect 115:1–14, 1995.
15. Faro S: Postpartum endometritis. In Pastorek JG II (ed): Obstetric and Gynecologic Infectious Disease. New York: Raven Press, pp 427–433, 1994.
16. Viglionese A, Nottebart VF, Bodman HA, Platt R: Recurrent group A streptococcal carriage in a health care worker associated with widely separated nosocomial outbreaks. Am J Med 91:329S–333S, 1991.
